# Authors’ Response to the Peer Review of “Estimating Variance of Log Standardized Incidence Ratios Assessing Health Care Providers’ Performance: Comparative Analysis Using Bayesian, Bootstrap, and Delta Method Approaches”

**DOI:** 10.2196/83796

**Published:** 2025-10-09

**Authors:** Solomon Woldeyohannes, Yomei Jones, Paul Lawton

**Affiliations:** 1 Menzies School of Health Research, Charles Darwin University, Northern Territory, Darwin, Casuarina, 0811, Australia, 61 0424635541; 2 School of Veterinary Sciences, University of Queensland, Gatton, Australia

**Keywords:** standardized incidence ratio, SIR, performance, health care provider, machine learning, equity

*This is the authors’ response to the peer-review report of “Estimating Variance of Log Standardized Incidence Ratios Assessing Health Care Providers’ Performance: Comparative Analysis Using Bayesian, Bootstrap, and Delta Method Approaches.”*

## Round 1 Review

### Reviewer MT [1]

#### General Comments

*This paper [2] compares 3 approaches for estimating the variance of the log standardized incidence ratio when profiling kidney replacement therapy centers in Australia: (1) the analytical delta method, (2) the nonparametric bootstrap method (5000 resamples), and (3) Bayesian Markov chain Monte Carlo (25,500 iterations, 3 chains). Using 2005‐2023 patient-level data from the Australia and New Zealand Dialysis and Transplant Registry and a random-effects logistic model, the authors evaluated bias, variance, and mean squared error (MSE) and visualized performance via funnel plots. Results indicated similar bias across methods but substantially lower variance and MSE for the Markov chain Monte Carlo method (bias≈0.019; variance=0.00005; MSE=0.00042) compared with the bootstrap method (variance=0.00027; MSE=0.00094).*

*The topic is practical and timely, yet the manuscript needs clearer model specification, interval coverage evaluation, and streamlined writing before it reaches publishable quality.*

**Response**: We sincerely appreciate Reviewer MT’s invaluable comments. We have substantially improved the content of the manuscript in response to the comments and questions of Reviewer MT. Also, thank you for acknowledging the practicality and timeliness of the presentation of our work. Herein, we have addressed point-by-point the concerns raised by Reviewer MT

### Specific Comments

#### Major Comments

*1. There are some concerns around model clarity (Poisson versus logistic language mixed; appendix is missing). Provide complete model equations, a covariate list, and software/code links and justify using the Bernoulli model for a ratio outcome.*

**Response:**

*(a) Poisson versus logistic language mixed.*

As a limitation of our study, we tried to indicate that other models—for instance, the use of the Poisson model for aggregated count data (provided we aggregated the data per center and per covariate for categorical predictors)—could affect the results of the variance estimates using the 3 methods. Hence, we have replaced logistic versus Poisson with the following statement: “First, while understanding the differences between variance estimation methods is crucial for assessing the reliability of standardized incidence ratio (SIR) estimates across different centers, we did not consider how model choice influences variance estimates and hence the resulting statistical inference. That is, we only used the hierarchical logistic regression model for modeling the binary individual-level outcome. Therefore, we did not explore the implication of using the Poisson model for aggregated data on the resulting variance estimates using the 3 methods.” Please see the Limitations section for this update. Given this limitation, we are planning future work that will compare Poisson and logistic models and determine the implications on variance estimates of using the 3 methods.

*(b) Appendix is missing.*

Thank you! We have now included the appendices and their citations. Please see the updated manuscript for the changes we have made.

*(c) Provide complete model equations.*

Below are the changes that we have made based on your suggestions for the model formula:

The model equation is specified as status ~ gender+agegp+indigenous+lung+diabetes+cvd+late+bmi30+mmm+timegp+(1|centre_id). For details of the update following your suggestion, please see the Methods section of the manuscript. We have provided the model equation. Details of the covariates included in the model equation are also described in the main manuscript.

As can be seen from the above equation, we have included the following covariates: age group, gender, Indigenous status, lung disease, diabetes, obesity, cardiovascular diseases (CVD), referral status, remoteness, and time period, which are defined as follows in our dictionary.

Variable code categories and explanations: gender=a binary variable with male versus female categoriesagegp=a categorical variable with 7 categories: ≥16‐26, ≥26‐36, ≥36‐46, ≥46‐56, ≥56‐66, ≥66‐76 and ≥76 years indigenous=Indigenous status with Indigenous versus non-Indigenous categorieslung=lung diseases (yes vs no)

diabetes=diabetes status (yes versus no)late=late referral status (yes versus no)bmi30=binary BMI status (BMI <30 vs BMI ≥30)mmm=Monash modified model remoteness scale with 7 categories (metropolitan areas [MM1], regional centers [MM2], large rural towns [MM3], medium rural towns [MM4], small rural towns [MM5], remote communities [MM6], and very remote communities [MM7])

timegp=time periods: 2012‐2015, 2016‐2019, and 2020-2023

*(e) Software/code link.*

We would be happy to include the R codes upon acceptance of our manuscript.

*(f) Justify using the Bernoulli model for a ratio outcome.*

*(d) Covariate list.*

- gender=a binary variable with male versus female categories
- agegp=a categorical variable with 7 categories: ≥16‐26, ≥26‐36, ≥36‐46, ≥46‐56, ≥56‐66, ≥66‐76 and ≥76 years
- indigenous=Indigenous status with Indigenous versus non-Indigenous categories
- lung=lung diseases (yes vs no)
- diabetes=diabetes status (yes versus no)
- late=late referral status (yes versus no)
- bmi30=binary BMI status (BMI <30 vs BMI ≥30)
- mmm=Monash modified model remoteness scale with 7 categories (metropolitan areas [MM1], regional centers [MM2], large rural towns [MM3], medium rural towns [MM4], small rural towns [MM5], remote communities [MM6], and very remote communities [MM7])
- timegp=time periods: 2012‐2015, 2016‐2019, and 2020-2023

As can be seen from the above equation, we have included the following covariates: age group, gender, Indigenous status, lung disease, diabetes, obesity, cardiovascular diseases (CVD), referral status, remoteness, and time period, which are defined as follows in our dictionary.

Variable code categories and explanations: gender=a binary variable with male versus female categoriesagegp=a categorical variable with 7 categories: ≥16‐26, ≥26‐36, ≥36‐46, ≥46‐56, ≥56‐66, ≥66‐76 and ≥76 years indigenous=Indigenous status with Indigenous versus non-Indigenous categorieslung=lung diseases (yes vs no)diabetes=diabetes status (yes versus no)late=late referral status (yes versus no)bmi30=binary BMI status (BMI <30 vs BMI ≥30)mmm=Monash modified model remoteness scale with 7 categories (metropolitan areas [MM1], regional centers [MM2], large rural towns [MM3], medium rural towns [MM4], small rural towns [MM5], remote communities [MM6], and very remote communities [MM7])

timegp=time periods: 2012‐2015, 2016‐2019, and 2020-2023

*(e) Software/code link.*

We would be happy to include the R codes upon acceptance of our manuscript.

*(f) Justify using the Bernoulli model for a ratio outcome.*

*(f) Justify using the Bernoulli model for a ratio outcome.*

We have included the following 2 paragraphs in the main manuscript to justify the Bernoulli model. Please see the Model Specification and Likelihood Definition section. “This approach is methodologically valid and commonly used in Bayesian hierarchical modeling and disease mapping, especially when individual-level data are available but aggregate counts are not directly observed. Modeling binary outcomes using Bernoulli likelihoods (ie, logistic regression) is appropriate for estimating probabilities of outcomes conditional on covariates. These estimated probabilities can then be summed within groups to yield expected counts for computing the SIR [standardized incidence ratio] or relative risks. This technique allows the derivation of the SIR from model-based expected counts, which is consistent with the definition of indirect standardization [3-7].”

Application studies using this approach include Kasza et al (2013) [8] and Normand et al (2007) [9]. The application of random intercept multilevel logistic regression models for indirectly standardizing performance measures was explored by Clark and Moore (2011) [10] using National Trauma Data Bank data for the admission year 2008. Zang et al (2013) [11] explored hierarchical logistic regression modeling under various conditions by applying Bayesian and frequentist methods.

Further details of the model specification can be seen in the appendices.

*2. Interval coverage and type I error absent. Action: add a simulation or internal bootstrap to report 95% interval coverage and false-positive rates for each method.*

**Response:** Thanks for the insight! We have now included 95% interval coverage for the bootstrapping and a credible interval for the Bayesian Markov chain Monte Carlo simulation results. Please see Table 2 for the update. We have also included abbreviations at the bottom of the table. We have included an additional table (Table 3) for the 95% false-positive rates.

*3. Missing data handling unexplained. Action: quantify missingness, describe any imputation, and list all risk-adjustment covariates.*

**Response:** We have added the following description of how the data and missingness are handled. For details, please see the Data Source and Management section. Accordingly, we have included the following two paragraphs:

“We received n=55,856 patient data on the course of treatments from February 14, 1992, to December 31, 2023. Since our initial study period was defined from January 1, 2005, to December 31, 2023, we excluded patient data from before January 1, 2005. This resulted in N=46,160 observations on the course of treatment and comorbidities data. With the revised study period definition (January 1, 2012, to December 31, 2023), following consultation with a team of chief investigators, a total of 11,586 observations were excluded (N=44,270). Due to 1743 missing observations for late referral, 808 on weight, and 188 on height variables, N=41,531 patient data were retained.

In addition, for comparison purposes, centers were split into Indigenous and non-Indigenous centers. Some centers had fewer than 20 Indigenous patients. This required considering the adequate count of Indigenous patients per center for running the hierarchical logistic regression. Accordingly, centers with fewer than 20 Indigenous patients were excluded, which resulted in n=16,243 (25,288 observations deleted) individual-level data. Moreover, we dropped patients with missing postcodes (2640 observations deleted), so that a total of N=13,603 remained. Finally, among the 13,603 observations, 3309 observations had censored status and were therefore excluded. In addition, we excluded 55 missing observations on lung diseases, cardiovascular diseases, and diabetes combined. Therefore, a total of 10,195 observations were included in our study.”

In addition, the Australia and New Zealand Dialysis and Transplant Registry indicated that data submission is complete though voluntary.

We have discussed this in the Data Source and Management subsection of the Methods section and added the two references listed here to support the quality of the data:

1. McDonald SP. Australia and New Zealand Dialysis and Transplant Registry. Kidney Int Suppl (2011). 2015 May 29;5(1):39‐44. doi: 10.1038/kisup.2015.8. PMID: 26097784.

2. ANZDATA Registry. 38th Report, Chapter 12: Indigenous People and End Stage Kidney Disease. Australia and New Zealand Dialysis and Transplant Registry. 2016. URL:https://www.anzdata.org.au/wp-content/uploads/2023/10/c12_anzdata_indigenous_v3.0_201600128_web.pdf. Accessed September 23, 2025.

#### Minor Comments

*For Table 1, add units and align decimals.*

**Response:** We have updated the Results section by adding the “natural log scale” at the bottom of each table. Also, we have changed the decimals to 3 digits for Table 2 and Table 3. We have left the decimals in Table 1 as is to show small differences among the methods on the variability per center.

## Abbreviations

CVD: cardiovascular disease
MSE: mean squared error
